# Active mechanistic target of rapamycin plays an ancillary rather than essential role in zebrafish CNS axon regeneration

**DOI:** 10.3389/fncel.2015.00251

**Published:** 2015-07-07

**Authors:** Heike Diekmann, Pascal Kalbhen, Dietmar Fischer

**Affiliations:** Division of Experimental Neurology, Department of Neurology, Heinrich-Heine-University of DüsseldorfDüsseldorf, Germany

**Keywords:** mTOR, optic nerve regeneration, zebrafish, rapamycin, pS6

## Abstract

The developmental decrease of the intrinsic regenerative ability of the mammalian central nervous system (CNS) is associated with reduced activity of mechanistic target of rapamycin (mTOR) in mature neurons such as retinal ganglion cells (RGCs). While mTOR activity is further decreased upon axonal injury, maintenance of its pre-injury level, for instance by genetic deletion of the phosphatase and tensin homolog (PTEN), markedly promotes axon regeneration in mammals. The current study now addressed the question whether active mTOR might generally play a central role in axon regeneration by analyzing its requirement in regeneration-competent zebrafish. Remarkably, regulation of mTOR activity after optic nerve injury in zebrafish is fundamentally different compared to mammals. Hardly any activity was detected in naïve RGCs, whereas it was markedly increased upon axotomy *in vivo* as well as in dissociated cell cultures. After a short burst, mTOR activity was quickly attenuated, which is contrary to the requirements for axon regeneration in mammals. Surprisingly, mTOR activity was not essential for axonal growth *per se*, but correlated with cytokine- and PTEN inhibitor-induced neurite extension *in vitro*. Moreover, inhibition of mTOR using rapamycin significantly reduced axon regeneration *in vivo* and compromised functional recovery after optic nerve injury. Therefore, axotomy-induced mTOR activity is involved in CNS axon regeneration in zebrafish similar to mammals, although it plays an ancillary rather than essential role in this regeneration-competent species.

## Introduction

The regenerative ability of the mammalian central nervous system (CNS) is dramatically reduced after birth, causing impaired regeneration of severed axons in adult animals. This regenerative failure is partly based on the limited intrinsic regenerative ability of mammalian CNS neurons and the inhibitory environment of axonal growth cones (Schwab and Bartholdi, 1996; Goldberg et al., 2002; Fischer and Leibinger, 2012). However, some degree of axonal regeneration is observed upon induction of cytokine expression by inflammatory stimulation (Fischer et al., 2000; Leibinger et al., 2009, 2013; Hauk et al., 2010), intravitreal application of ciliary neurotrophic factor (CNTF) (Lingor et al., 2008; Müller et al., 2009) and virus-mediated cytokine expression (Leaver et al., 2006; Pernet et al., 2013). In addition, genetic manipulation of certain signaling cascades, such as phosphatidylinositol 3-kinase/protein kinase B/mechanistic target of rapamycin (PI3K/AKT/mTOR) and Janus kinase/signal transducer and activator of transcription (JAK/STAT) have been described to promote axon regeneration (Cai et al., 2001; Gao et al., 2004; Fischer and Leibinger, 2012; Diekmann and Fischer, 2013; Pernet et al., 2013). In particular, genetic deletion of the phosphatase and tensin homolog (PTEN), an opponent of PI3K, potently promotes axon regeneration of RGCs, motor, corticospinal and peripheral neurons (Park et al., 2008; Christie et al., 2010; Liu et al., 2010; Ning et al., 2010). In RGCs, these effects were, for the most part, mimicked by knockout of tuberous sclerosis complex 1 (TSC1), a negative regulator of mTOR signaling (Park et al., 2008). Therefore, the growth promoting effects of PTEN deletion have been mainly attributed to sustained activity of downstream mTOR. This serine-threonine protein kinase is central to a multi-functional signaling network that integrates intra- and extracellular signals to control cell proliferation, metabolism and survival (Maiese et al., 2013). Upon nucleation into multi-protein complexes, mTOR phosphorylates, among others, ribosomal protein S6 kinase 1 (S6K1) and eukaryotic initiation factor 4E-binding protein 1 (4E-BP1), thereby promoting anabolic processes such as protein and lipid synthesis (Laplante and Sabatini, 2009). Inhibition of mTOR by rapamycin attenuates most of the beneficial effects of PTEN deletion in RGCs, compromises optic nerve regeneration upon inflammatory stimulation and partially blocks the conditioning effect of sciatic nerve injury (Park et al., 2008; Abe et al., 2010; Leibinger et al., 2012). Therefore, these findings suggest that mTOR plays a central role in regenerative processes of injured axons in mammals and raise the question as to whether mTOR activity is also required for axon growth stimulation in regeneration-competent animals.

In contrast to mammals, adult teleosts have the remarkable ability to functionally regenerate injured CNS axons (Stuermer et al., 1992; Bernhardt et al., 1996; Becker and Becker, 2007). Upon crush or complete transection of the optic nerve, RGCs survive and regrow axons through the optic nerve and tract to topographically re-innervate their respective targets in the brain, leading to functional restoration of the visual projection within 2–4 weeks after injury (Stuermer et al., 1992; Bernhardt, 1999; McDowell et al., 2004). We recently established the adult transgenic growth associated protein 43 (GAP43):green fluorescent protein (GFP) zebrafish as a valuable tool for axon regeneration studies, as they strongly express GFP only in injured neurons and their regrowing axons (Diekmann et al., 2015). In addition, the analysis of regenerating axons was facilitated using a combination of wholemount optic nerve tissue clearing and confocal microscopy (Diekmann et al., 2015). Taking advantage of these novel approaches, we here investigated the regulation of mTOR after optic nerve injury in adult zebrafish and whether manipulation of mTOR activity would impact RGC axon regeneration *in vitro* and *in vivo*. Indeed, we detected pronounced, although transient increase of mTOR activity in most RGCs upon optic nerve injury *in vivo* as well as in dissociated cell cultures. Moreover, inhibition of mTOR attenuated CNTF- and PTEN inhibitor-induced, but not basal axonal growth *in vitro* as well as axon regeneration *in vivo*. In addition, functional recovery *in vivo* was significantly compromised upon rapamycin treatment, indicating a contribution of mTOR signaling to spontaneous axon regeneration in zebrafish.

## Materials and Methods

### Zebrafish

Adult, 4–9 months old wild-type or homozygous Tg(GAP43:GFP) zebrafish (Udvadia, 2008), in the text referred to as GAP43:GFP, were used for all experiments. Zebrafish were reared and kept on a 14 h light/ 10 h dark cycle under standard conditions (Westerfield, 1989). All experimental procedures were approved by the local animal welfare committee in Recklinghausen and conducted in compliance with federal and state guidelines for animal experiments in Germany.

### Dissociated Retinal Cell Cultures

Tissue culture plates (24- or 96-well-plates; Nunc) were coated with poly-D-Lysine (0.1 mg/ml, molecular weight 3,00,000 Da; Sigma) for 30 min at 37°C, rinsed twice with distilled water and air-dried. Zebrafish were sacrificed by immersion in MS222 (0.4 mg/l) and decapitation. Retinae were rapidly dissected from the eyecups of naïve zebrafish and incubated in a digestion solution containing papain (10 U/ml, Worthington) and L-cysteine (0.3 μg/ml; Sigma) in L15/salt solution (12.5% salt solution: 10 mM D-glucose, 1.26mM CaCl_2_, 32 mM Hepes, pH 7.5/ 87.5% L15; Invitrogen) at room temperature for 40 min. They were then rinsed with L15/salt solution and triturated in 2 ml fish medium (2% FBS (Invitrogen), 0.2 mg/ml penicillin/streptomycin (Biochrom) in L15/salt solution). Dissociated cells were passed through a cell strainer (40 μm; Falcon) and counted using a TC10 Automated Cell Counter (BioRad). Approximately 1.5 × 10^4^ cells (96 well plate) and 2.5 × 10^4^ cells (24 well plate) were added to each well, respectively. In some wells, 1 ng/ml mouse CNTF (Peprotech), 10 nM rapamycin (LC Laboratories) or 10 nM bisperoxovanadium (bpV(phen), Calbiochem) were added to the culture medium. Cultures were incubated at 27.5 °C in a humidified incubator for 2 h to 4 days. Cells were fixed with 4% paraformaldehyde (PFA) in phosphate-buffered saline (PBS) for 30 min at room temperature and permeabilized with Methanol (5 min) for immunostaining. Neurite growth was determined after 4 days in culture. RGCs with regenerated neurites were visualized via GFP expression or acetylated tubulin antibody (1:2,000; Sigma) immunostaining and photographed under a fluorescent microscope (200×, Observer.D1, Zeiss). Neurite length was determined using ImageJ software as previously described (Diekmann et al., 2015). Mean neurite length was calculated by dividing the sum of neurite length by the number of RGCs with regenerated neurites per well. Data are given as means ± SEM of at least six replicate wells from at least two independent experiments. The significance of intergroup differences was evaluated using Two-Way Anova with Holm-Sidak *post hoc* tests (GraphPad; SigmaStat).

### Optic Nerve Crush

For surgery, zebrafish were anesthetized by immersion in MS222 (0.18 mg/l; Sigma). The eye was slightly pulled out of its orbit to expose the optic nerve. Taking care to spare the ophthalmic artery, the optic nerve was intra-orbitally crushed ~0.5 mm behind the eye for 5 s using jeweler’s forceps (FST), as described previously (Bormann et al., 1999; Liu and Londraville, 2003; Diekmann et al., 2015).

### *In vivo* Rapamycin Treatment

A rapamycin stock of 1 mg/ml was prepared in DMSO. Immediately after optic nerve crush, fish were placed into 0.2 μM rapamycin (1:5,000 dilution of stock in fish water) or 0.02% DMSO as control. Solutions were changed daily.

### Retinal Cross Sections

Zebrafish were sacrificed by prolonged immersion in MS222 (0.4 mg/l) and decapitation. Eye(s) were removed and fixed in 4% PFA/PBS at 4°C overnight. Subsequently, eyes were immersed in 30% sucrose and embedded in Tissue-Tek (Sakura). Frozen sections (14 μm) were cut on a CM3050S cryostat (Leica), thaw-mounted onto glass slides (Superfrost plus; ThermoFisher) and stored at −80°C until further use.

### Immunohistochemistry

Retinal cross sections were permeabilized with 100% Methanol for 5 min at room temperature. After blocking with 2% BSA/5% donkey serum/PBS, they were incubated with either pS6(Ser235/236) (1:500; Cell Signaling #4857), choline acetyl transferase (CHAT; 1:100; Millipore AB144P) or acetylated tubulin (1:1,000; Sigma T6793) antibodies overnight at 4°C. The protein sequence of zebrafish ribosomal S6 around the detected phosphorylation site is 100% identical to the human peptide used for antibody generation. After several washes with PBS, bound antibodies were visualized with anti-mouse or anti-goat secondary antibodies conjugated to Alexa Fluor 488 or Alexa Fluor 594 (1:1,000; Molecular Probes). Fluorescent sections were embedded in Mowiol and analyzed under a fluorescent microscope (200×, Observer.D1, Zeiss). The percentage of pS6-positive cells was calculated in relation to the number of cells stained with acetylated tubulin in the ganglion cell layer (RGCs) and CHAT-positive cells in the inner nuclear layer (INL; amacrines), respectively, on six sections from 2–3 different zebrafish at each time point.

### Quantification of Axon Regeneration

Two and a half days after optic nerve crush, zebrafish were sacrificed by prolonged immersion in MS222 (0.4 mg/l) and decapitation. The lower jaw and gills were removed and the eyes pulled slightly out of their sockets to stretch the optic nerves. The head was then fixed in 75 mM Lysine/2% PFA/10 mM NaIO_4_ overnight at 4°C. After fixation, the optic nerves were dissected with part of the retina attached and placed into FocusClear solution (BioRad) overnight for clearing. They were embedded in MountClear and scanned using a confocal microscope (LSM510, Zeiss). For quantification of axon regeneration, axon profiles within the nerve diameter were counted on five individual z-sections at 200 and 500 μm distal from the lesion site, respectively. “Axons per mm” was calculated by dividing the number of axons per site by the diameter of the nerve of the respective z-section. Data are given as means ± SEM of eight optic nerves per group from two independent experiments. The significance of intergroup differences was evaluated using Two-Way ANOVA with Holm-Sidak *post hoc* tests (GraphPad; SigmaStat).

### Dorsal Light Reaction

Fish swim in a slightly oblique position (~10°) upon unilateral optic nerve injury (Figure 5E; Lindsey and Powers, 2007; Mensinger and Powers, 2007), which is gradually reversed with ongoing regeneration. Therefore, the degree of tilt can serve as a measure for functional regeneration. At various times after optic nerve injury, fish were placed into a 2.7 × 17.5 cm container with 400 ml water. After ~5 min adaptation, they were recorded on video for 1–2 min, making sure to capture at least five straight swims directly towards the camera. The videos were analyzed frame by frame and still pictures taken if the whole body of the fish was positioned straight towards the camera. The angle between the fish body position (straight line through the eyes; Figure 5E) and the horizon was then determined using ImageJ. At least seven different pictures were analyzed per fish and time point to calculate the mean divergent angle. Data are given as means ± SEM of at least five fish per group. The significance of intergroup differences was evaluated using Repeat-Measurements Two-Way ANOVA with Holm-Sidak *post hoc* tests (GraphPad; SigmaStat).

### RNA Isolation and Quantitative Real-Time PCR

For quantitative real-time PCR, both retinae from one zebrafish were dissected and combined. Total RNA was isolated using the RNeasy Mini kit (Qiagen, Germany) according to the manufacturer’s instructions, including the removal of genomic DNA. Total RNA (300 ng) was reverse transcribed using the SuperScript II reverse transcriptase kit (Invitrogen, USA). Gfp, gap43 and glyceraldehyde 3-phosphate dehydrogenase (gapdh) were amplified using SYBR Green PCR Master Mix (Applied Biosystems, Foster City, CA, USA) and primers gfp-for (5′-GCAAGCTGACCCTGAAGTTC-3′), gfp-rev (5′-GGTGCGCTCCTGGACGTA-3′), gap43-for (5′-TGCTGCATCAGAAGAACTAA-3′), gap43-rev (5′-CCTCCGGTTTGATTCCATC-3′), gapdh-for (5′-CCTCCGGTTTGATTCCATC-3′) and gapdh-rev (5′-GGCGGTGTAGGCATGAAC-3′) on an Applied Biosystems 7500 real-time PCR system (Life Technologies) using 45 amplification cycles according to the manufacturer’s protocol. PCR efficiency was confirmed for each primer pair and specificity verified with the dissociation curve analysis feature. Gfp and gap43 mRNA expression levels in rapamycin-treated vs. control retinae were quantified using the ΔΔCt method (Livak and Schmittgen, 2001) with gapdh as the reference gene. All reactions were performed in triplicate and at least three independent samples (from different zebrafish) were analyzed per experimental group.

## Results

### Optic Nerve Injury Induces mTOR Activity in Zebrafish Retinae

**Figure 1:**
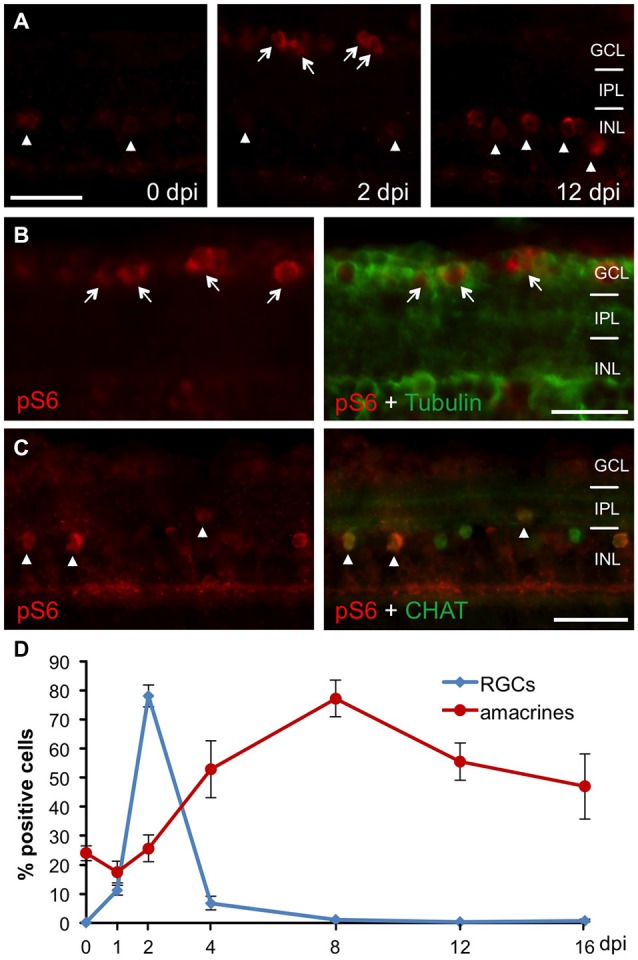
**Retinal mTOR activation upon optic nerve injury. (A)** Immunohistochemistry of phosphorylated S6 (pS6) on retinal cross sections at 0, 2 and 12 days post injury (dpi) indicates induction of mTOR activity in the ganglion cell (GCL, arrows) and inner nuclear (INL, arrowheads) layers after optic nerve axotomy. **(B)** Co-immunostaining of a retinal cross section (2 dpi) with pS6 (red) and acetylated tubulin (green) antibodies identifies pS6-positive cells as retinal ganglion cells (RGCs; arrows). **(C)** Co-immunostaining of a retinal cross section (0 dpi) with pS6 (red) and choline acetyl transferase (CHAT, green) antibodies identifies cholinergic amacrine cells (arrowheads). **(D)** Time course of pS6-positive RGCs (blue) and amacrines (red) after optic nerve crush. Data represent means ± SEM of two independent experiments. GCL = ganglion cell layer; IPL = inner plexiform layer; INL = inner nuclear layer. Scale bar = 25 μm.

In order to analyze mTOR activity upon optic nerve regeneration in zebrafish, we examined the spatiotemporal distribution of phosphorylated ribosomal protein S6 (pS6), a downstream effector of mTOR, on retinal cross sections at various times after optic nerve injury (Figure 1). In naïve, uninjured zebrafish retinae, pS6 immunostaining was detected only in a few cells of the INL (Figure 1A). In comparison, S6 phosphorylation was quickly, although transiently induced in acetylated tubulin-positive RGCs upon optic nerve crush (Figures 1A,B,D). At 2 days post injury (dpi), 78.1 ± 3.8% RGCs were strongly positive for pS6, whereas hardly any pS6 signal was detected in RGCs at time points beyond 4 dpi (Figure 1D). Interestingly, injury-induced mTOR activation was also observed in cholinergic amacrines identified by co-staining with antibodies against cholinergic acetyl transferase (CHAT) in the INL (Figures 1A,C,D). However, pS6 immunoreactivity in amacrines was only significantly increased at 4–16 dpi. Thus, optic nerve injury induced mTOR activation in RGCs and amacrine cells with different time courses.

### mTOR Activity is Induced in Cultured RGCs

**Figure 2:**
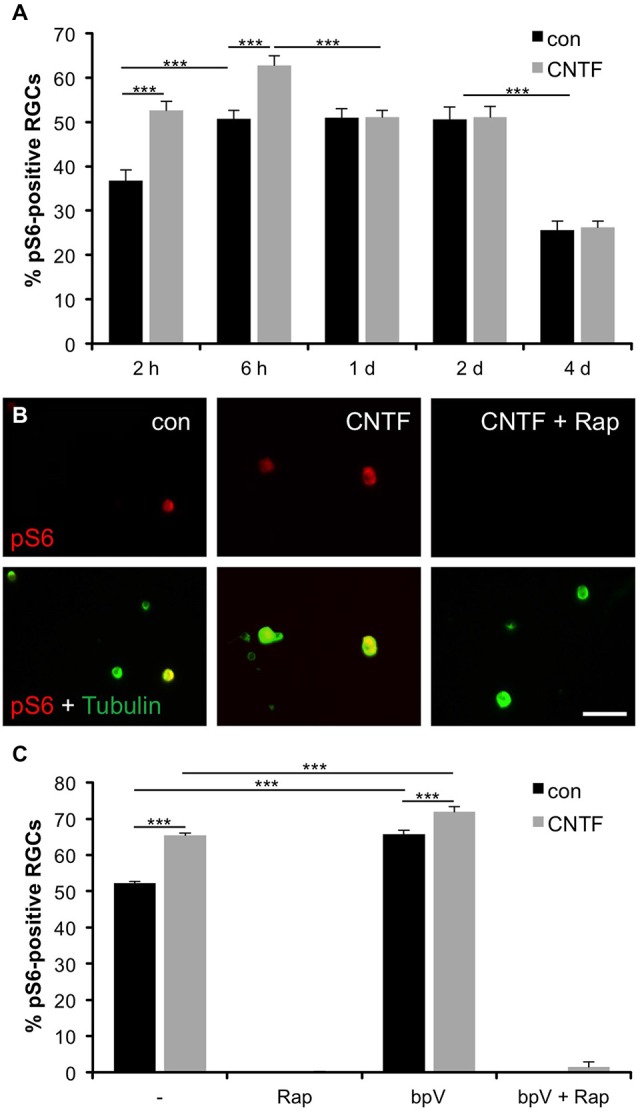
**Induction of mTOR activity in cultured RGCs. (A)** Quantification of the percentage of retinal ganglion cells (RGCs) positive for phosphorylated S6 (pS6) at 2 and 6 hours (h) as well as 1, 2 and 4 days (d) in culture with or without ciliary neurotrophic factor (CNTF; 1 ng/ml) treatment. Data represent means ± SEM of three independent experiments, each with three repeats. Treatment effects: *p* < 0.001. **(B)** Representative pictures of cultured RGCs stained with pS6 (red) and acetylated tubulin (green) antibodies at 6 h in culture. Cells were either treated with vehicle (con), CNTF (1 ng/ml) or CNTF + rapamycin (Rap; 10 nM). Scale bar = 50 μm. **(C)** Percentage of pS6-positive RGCs at 6 h in culture after treatment with either vehicle (−), rapamycin (Rap; 10 nM), PTEN inhibitor bisperoxovanadium (bpV(phen); 10 nM), or bpV(phen) + Rap. Data represent means ± SEM of at least two independent experiments, each with three repeats. Treatment effects: *p* < 0.001.

To test whether mTOR is also activated in axotomized RGCs *in vitro*, we co-stained dissociated retinal cultures from naïve zebrafish with pS6 and acetylated tubulin antibodies at various time points. At 2 h in culture, already 36.7 ± 2.5% RGCs were pS6-positive (Figure 2A). This proportion rose to 50.8 ± 1.9% by 6 h and stayed at this level for 2 days before significantly declining at 4 days in culture (25.7 ± 2.4%). Therefore, mTOR activity was similarly induced in axotomized RGCs in culture and *in vivo*. The faster onset and lower overall penetrance (~50 vs. ~80%; compare Figures 1D, 2A) is likely attributable to the cell culture conditions.

To assess whether mTOR activity could be modulated by cytokines similar to mammalian RGCs (Park et al., 2008; Leibinger et al., 2012), some retinal cultures were concurrently treated with CNTF. CNTF has recently been shown to promote neurite growth of zebrafish RGCs (Elsaeidi et al., 2014; Diekmann et al., 2015) and indeed significantly increased the percentage of pS6-positive RGCs at 2 and 6 h compared to controls (52.6 ± 2% and 62.8 ± 2.1%, respectively) (Figures 2A,B). However, no CNTF-induced increase of mTOR activity was observed at 1, 2 and 4 days in culture (Figure 2A). Interestingly, addition of CNTF to retinal cultures at 1 day post dissociation could similarly not promote increased pS6 levels (data not shown), indicating insensitivity to CNTF at later incubation times.

We also validated that mTOR activation was indeed the underlying cause of S6 phosphorylation in zebrafish RGCs. Application of the potent mTOR inhibitor rapamycin abolished S6 phosphorylation in RGCs, independent of CNTF treatment (Figures 2B,C). Conversely, the PTEN inhibitor bpV(phen), which promotes PI3K and subsequently mTOR activity (Posner et al., 1994; Schmid et al., 2004), significantly increased the percentage of pS6-positive RGCs either alone or in combination with CNTF compared to untreated controls (65.7 ± 1.1% (bpV) and 71.9 ± 1.4% (bpV + CNTF) vs. 52.2 ± 0.4% (control); Figure 2C). Similar to CNTF treatment, bpV(phen) was unable to increase mTOR activity upon application at 1 day in culture (data not shown). Concurrent bpV(phen) + rapamycin treatment again annulled S6 phosphorylation (Figure 2C), indicating that bpV(phen) acts on S6 through increasing mTOR activity. Overall, the extent of S6 phosphorylation correlates with mTOR activity in zebrafish RGCs.

### Induction of Neurite Growth Correlates with mTOR Activity

**Figure 3:**
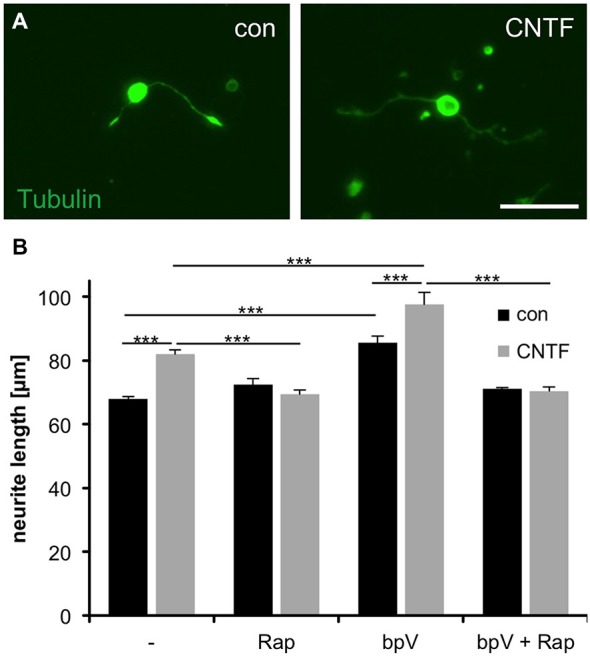
**Correlation of mTOR activity with CNTF- and PTEN inhibitor-induced neurite growth of cultured RGCs. (A)** Representative pictures of cultured retinal ganglion cells (RGCs) stained with acetylated tubulin antibody at 4 days in culture and exposure to vehicle (con) and ciliary neurotrophic factor (CNTF, 1 ng/ml), respectively. Scale bar = 50 μm. **(B)** Quantification of average RGC neurite length at 4 days in culture after treatment with either vehicle control (−), rapamycin (Rap; 10 nM), PTEN inhibitor bpV(phen) (10 nM), or bpV(phen) + Rap. Data represent means ± SEM of at least two independent experiments, each with three repeats. Treatment effects: *p* < 0.001.

In an effort to evaluate whether mTOR activity is required for axonal growth, we next measured neurites of isolated RGCs grown in the presence of rapamycin (mTOR inhibition) or bpV(phen) (mTOR activation). Interestingly, rapamycin concentrations abolishing S6 phosphorylation (Figures 2B,C) did not affect neurite growth *per se* [72.4 ± 1.9 μm (Rap) vs. 68.0 ± 0.8 μm (untreated control)]. However, treatment with either CNTF or bpV(phen), which increased mTOR activity to comparable extents, significantly increased neurite growth of dissociated RGCs (82.0 ± 1.3 μm and 85.6 ± 2.2 μm, respectively; Figures 3A,B). Moreover, combination of CNTF and bpV(phen), which increased mTOR activity the most (Figure 2C), also showed longer outgrowth than each treatment alone (97.6 ± 3.8 μm). These growth-promoting effects of CNTF and bpV(phen) were abrogated in the presence of rapamycin [69.4 ± 1.4 μm (CNTF + Rap), 71.1 ± 0.4 μm (bpV + Rap) and 70.5 ± 1.3 μm (CNTF + bpV + Rap)], indicating a correlation between increased mTOR activity and PI3K/AKT-stimulated neurite growth in culture (Figure 3B). Thus, mTOR activity is seemingly not required for basal axon growth, but enhanced levels are sufficient to promote further growth of RGC neurites in culture.

### Inhibition of mTOR Activity Compromises Axon Regeneration and Functional Recovery

**Figure 4:**
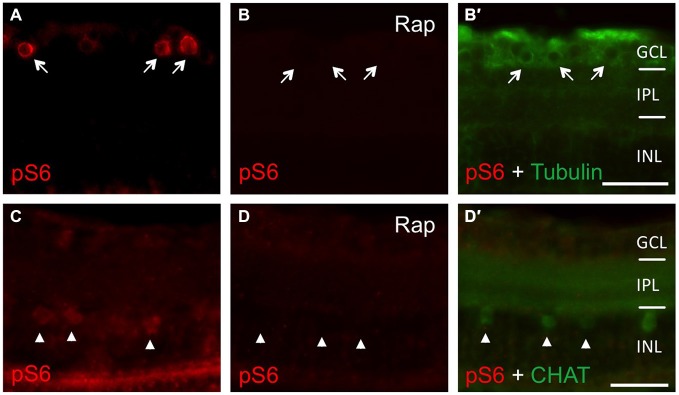
**Efficient inhibition of mTOR activity in zebrafish *in vivo*. (A, B, B′)** Immunostaining of retinal cross sections with phosphorylated S6 (pS6, red) and acetylated tubulin (green) antibodies at 2 days post injury. Fish were either treated with DMSO **(A)** or 0.2 μM rapamycin (Rap, **B**) prior to tissue preparation. Rapamycin treatment abrogated pS6 staining in retinal ganglion cells (RGCs; arrows). **(C,D,D′)** Immunostaining of retinal cross sections with pS6 (red) and choline acetyl transferase (CHAT; green) antibodies at 6 days post injury. Fish were either treated with DMSO **(C)** or 0.2 μM rapamycin (Rap, **D**) prior to tissue preparation. Rapamycin treatment abolished pS6 staining in amacrines (arrowheads). GCL = ganglion cell layer; IPL = inner plexiform layer; INL = inner nuclear layer; Scale bar = 25 μm.

In an attempt to investigate the relevance of mTOR activity for axonal regeneration, we first explored pharmacological approaches for efficient mTOR inhibition *in vivo*. In our hands, neither intravitreal nor intraperitoneal injections of rapamycin yielded consistent, longer-term down-regulation of retinal S6 phosphorylation (data not shown). Therefore, zebrafish were placed directly into either DMSO- or rapamycin-containing water immediately after optic nerve crush, as similar treatment has previously been shown to impede mTOR induction upon fin amputation (Hirose et al., 2014). Using this approach, mTOR activation was efficiently inhibited as verified by abolished pS6 levels on retinal cross-sections (Figure 4). Whereas S6 phosphorylation was strongly induced in RGCs of control retinae at 2 dpi (Figure 4A) and in cholinergic amacrines at 6 dpi (Figure 4C), no pS6 staining was detected in rapamycin-treated zebrafish at these time points (Figures 4B,D). Therefore, mTOR activity also underlies increased S6 phosphorylation upon optic nerve injury *in vivo* and can be prevented longer-term by supplementing the fish water with rapamycin.

**Figure 5:**
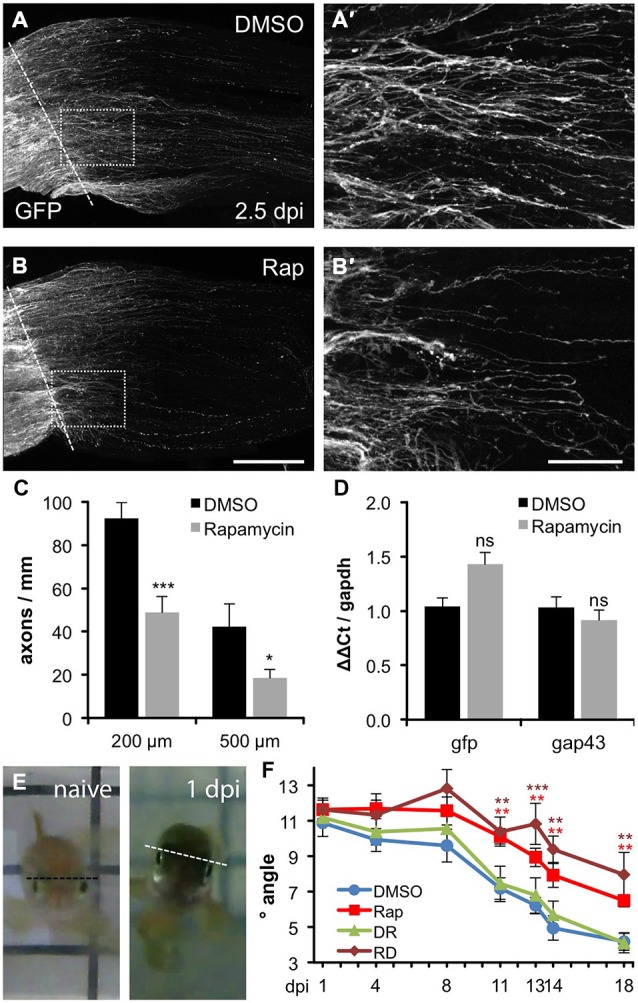
**Compromised axon regeneration and functional recovery upon mTOR inhibition. (A,B)** Maximum intensity projections (85 × 0.9 μm confocal z-sections) of wholemount optic nerves from DMSO- **(A)** and 0.2 μM rapamycin (Rap, **B**) -treated GAP43::GFP zebrafish at 2.5 days post injury (dpi). The lesion site is indicated with a dashed line, proximal is to the left. Scale bar = 200 μm. **(A′,B′)** Higher magnifications of the boxed areas in **(A)** and **(B)**, respectively, using maximum intensity projections of 10 × 0.9 μm confocal stacks. Scale bar = 50 μm. **(C)** Quantification of axon profiles per mm optic nerve diameter on single z-sections at 200 and 500 μm posterior to the lesion site of DMSO- and rapamycin-treated zebrafish, respectively (for details see “Materials and Methods” Section). Data represent means ± SEM of 8 optic nerves from two independent experiments. Treatment effects compared to DMSO control: ***p < 0.001; *p < 0.05 **(D)** Quantitative real-time PCR for green fluorescent protein (gfp) and growth associated protein 43 (gap43) in relation to glyceraldehyde-3-phosphate dehydrogenase (gapdh) in retinae isolated from zebrafish 2 days post injury that were treated either with vehicle (DMSO) or 0.2 μM rapamycin (Rap), respectively. Data represent mean ΔΔCt ± SEM of at least three different fish per experimental group. ns = non-significant **(E)** Representative pictures of the swimming position of a naïve zebrafish and a fish 1 day post unilateral right optic nerve crush (1 dpi), respectively. **(F)** Quantification of the oblique swimming position of DMSO (blue)- and rapamycin (Rap; red)-treated zebrafish at 1, 4, 11, 13, 14 and 18 days post injury (dpi). In addition, some zebrafish received rapamycin-treatment only during the first 3 days of the experiment (RD; brown). Another group was initially held in DMSO (0–3 dpi) and then transferred to rapamycin for the remainder of the experiment (DR; green). Data represent means ± SEM of at least five zebrafish per group. Treatment effects compared to DMSO control: ***p < 0.001; **p < 0.01.

To investigate whether inhibition of mTOR activity would impact axon regeneration upon optic nerve injury, we took advantage of our recently established protocol that enables the accurate analysis of regenerating axons in wholemount optic nerve preparations using GAP43:GFP transgenic zebrafish (Diekmann et al., 2015). As previously described, quite a few severed axons had already grown past the lesion site back into the injured nerve at 2.5 dpi in control animals (Figures 5A,A′). In comparison, rapamycin treatment reduced the number and/or length of regenerating axons as quantified at two different sites posterior to the lesion (Figures 5B,B′,C). Similarly, pixel intensities were reduced in optic nerves of rapamycin-treated zebrafish at 5 dpi (data not shown). Systemic rapamycin treatment has previously been described to reduce GAP43 expression in peripheral, but not optic nerves in mice (Abe et al., 2010; Leibinger et al., 2012). Therefore, we next addressed the possibility that the observed reduction of regenerating axons was rather based on impaired GFP expression. To this end, we compared transgenic gfp and endogenous gap43 mRNA expression levels in vehicle- and rapamycin-treated retinae by quantitative RT-PCR (Figure 5D). In comparison to control animals, neither gfp nor gap43 expression was significantly changed at 2 dpi in rapamycin-treated retinae, thereby excluding this possibility.

Finally, we investigated whether rapamycin-induced impairment of axonal regeneration would have functional consequences. As reported previously, zebrafish swim slightly oblique towards the side of the intact eye upon unilateral optic nerve crush (Figure 5E). This tilt lessens progressively with ongoing axonal regeneration (Lindsey and Powers, 2007; Mensinger and Powers, 2007). Thus, the swimming position can be used as an indicator for functional recovery of vision. In the vehicle-treated control group, significant improvements were initially detected at 11 dpi (7.2° vs. 10.9° at 1 dpi) and the swimming angle lessened to 4.2° at 18 dpi. In comparison, rapamycin-treated zebrafish swam in a significantly more tilted position from 11 dpi (10.1°) up to 18 dpi (6.5°; Figure 5F). Thus, impairment of mTOR activity upon optic nerve injury significantly compromised axonal regeneration and functional recovery. Interestingly, reduced regeneration was also observed upon temporary rapamycin treatment early after optic nerve crush (0–3 dpi), correlating with increased mTOR activity in RGCs (Figure 5F). On the other hand, late rapamycin application (4–18 dpi) had no effect on visual recovery, indicating that the delay of functional recovery was caused by abolished mTOR activation in RGCs within the first days after injury and that mTOR induction in cholinergic amacrines at later time points is negligible for optic nerve regeneration. Overall, mTOR signaling seems not absolutely essential for axonal regeneration, as rapamycin could only delay, rather than completely block axon growth and functional recovery. Nevertheless, the initial burst of mTOR activity upon optic nerve injury, which is fundamentally different to its regulation in mammals, supports regenerative processes in regeneration-competent zebrafish.

## Discussion

The current study characterizes the relevance of active mTOR for axonal regrowth and functional recovery after optic nerve injury in a regeneration-competent CNS model. Indeed, mTOR activity was markedly, although temporarily induced in zebrafish RGCs upon optic nerve injury *in vivo* as well as in dissociated neuronal cell cultures. However, mTOR activity was not essential for axonal growth *per se*, but correlated with cytokine- and PTEN inhibitor-induced neurite extension *in vitro*. Moreover, inhibition of mTOR using rapamycin significantly reduced axonal regeneration *in vivo* and compromised functional recovery after optic nerve injury. Thus, regulation of mTOR activity after axonal injury in regeneration-competent zebrafish seems fundamentally different from the non-regenerating mammalian CNS. Nevertheless, active mTOR contributes to axon regeneration in both systems.

Induction of mTOR in zebrafish retina upon optic nerve injury was monitored using the phosphorylation levels of ribosomal protein S6 as described previously (Park et al., 2008; Leibinger et al., 2012). S6 is phosphorylated by S6K1, which itself is a target of mTOR. Accordingly, rapamycin as an established mTOR inhibitor diminished pS6 levels in retinal cell cultures as well as *in vivo*. In addition, stimulation of mTOR through the PI3K/AKT pathway with either the cytokine CNTF or the PTEN inhibitor bpV(phen) consistently elevated pS6 levels in dissociated RGCs. Therefore, the phosphorylation status of S6 can be used as a faithful readout of mTOR activity in zebrafish retinae.

**Figure 6:**
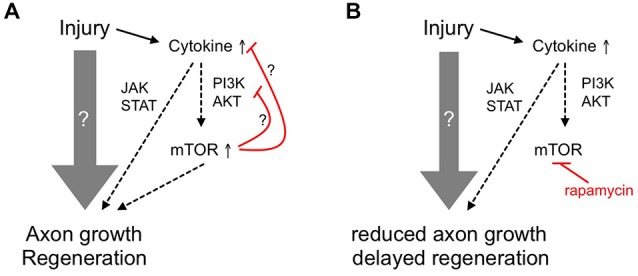
**Schematic showing the ancillary contribution of mTOR activity to axon regeneration in adult zebrafish. (A)** Upon optic nerve injury, diverse and prevalently unknown molecular mechanisms (gray arrow) are activated in adult zebrafish RGCs to enable axon growth and functional regeneration. Among others, cytokines such as LIF are released and activate the JAK/STAT and PI3K/AKT/mTOR pathways that contribute to axon regeneration. The initial burst of mTOR activity could then be quickly attenuated by negative feedback loops (red lines). **(B)** Inhibition of mTOR using rapamycin only partially reduces axon regrowth and compromises functional recovery, suggesting an ancillary rather than essential role of mTOR activity in zebrafish optic nerve regeneration.

The progression of mTOR activity after optic nerve injury in zebrafish differed significantly from the one in rodents. In retinae from naïve, adult mice, only 10–15% of RGCs contain high levels of phosphorylated S6 and reportedly have the highest probability to regenerate (Duan et al., 2015). This proportion is even further reduced upon optic nerve injury (Park et al., 2008; Leibinger et al., 2012). In contrast, no pS6 staining was observed in RGCs of naïve zebrafish retinae. However, S6 phosphorylation in RGCs was markedly induced *in vivo* shortly after optic nerve injury as well as in dissociated cell cultures. In addition, prolonged mTOR induction was observed in cholinergic amacrines after an initial lag phase. The activity of mTOR is reportedly controlled by the PI3K/AKT, MAPK/ERK, CDK42/phopholipase D and wnt pathways (Fang et al., 2001, 2003; Ma et al., 2005; Huang and Manning, 2009; Hirose et al., 2014). Interestingly, leukemia inhibitory factor (LIF), a IL6-like cytokine, which reportedly activates the PI3K/AKT/mTOR pathway in rodents (Li et al., 2014), is strongly upregulated in zebrafish RGCs early after axotomy (Ogai et al., 2014) and could potentially contribute to the transient mTOR activation (Figure 6). However, the elucidation of the detailed stimuli involved in activating mTOR after optic nerve injury in zebrafish awaits further investigations.

The burst of mTOR activity in RGCs shortly after optic nerve injury was quickly attenuated, as hardly any pS6-positive RGCs were detected at 4 dpi. This again approximately coincides with the attenuation of LIF expression in zebrafish (Ogai et al., 2014). In addition, activated mTOR itself has been shown to strongly repress the PI3K/AKT pathway upstream of PI3K, thereby forming a negative feedback loop (Harrington et al., 2005; Manning and Cantley, 2007). Furthermore, S6K1-mediated phosphorylation of Rictor inhibits mTORC2 and AKT signaling (Julien and Roux, 2010). Both of these auto-regulatory mechanisms could be involved in the observed mTOR down-regulation in zebrafish RGCs (Figure 6A). Interestingly, mTOR activity could not be induced by CNTF or PTEN inhibitor application at later time points in culture, indicating potential deactivation of this signaling pathway during later regenerative processes. For this reason, augmentation of *in vivo* mTOR activity with the PTEN inhibitor in an attempt to experimentally increase axon regeneration is envisaged impractical.

Overall, the observed time course of S6 phosphorylation matches the regulation of mTOR activity after injury in another regeneration competent model, the mammalian sciatic nerve. Two-fold increased pS6 levels were detected in dorsal root ganglion (DRG) neurons at 1 dpi that returned to basal levels by 4 dpi (Abe et al., 2010). Comparably, S6K phosphorylation was also transiently induced in regenerating blastemal cells 6 h to 3 days after zebrafish fin amputation (Hirose et al., 2014), consistently indicating short bursts of mTOR activity in regenerating tissues. In contrast, impaired regeneration in the mammalian CNS is associated with reduced mTOR activity in mature neurons, which is further decreased upon axonal injury (Park et al., 2008; Leibinger et al., 2012). Therefore, mTOR regulation in regeneration-competent (mammalian PNS, zebrafish) and -incompetent (mammalian CNS) systems seems fundamentally different and it would be interesting to analyze the effect of a comparable course of mTOR activity (short burst of strong mTOR activity in all RGCs followed by quick down-regulation) on the regenerative ability of mammalian CNS neurons.

Similar to mouse RGCs, basal neurite growth in cell culture was not affected by rapamycin treatment in zebrafish, as pS6-negative RGCs grew neurites equally well (Leibinger et al., 2012 and this study). On the other hand, mTOR activity closely correlated with PI3K/AKT-induced axonal growth of zebrafish RGCs. Consistent with previous reports (Elsaeidi et al., 2014; Diekmann et al., 2015), application of the cytokine CNTF or the PTEN-inhibitor pbV(phen) increased pS6 levels as well as neurite length in culture and this growth-promotion was abrogated in the presence of rapamycin. In mice RGCs, on the contrary, rapamycin could only compromise the neurite growth promoting effect of CNTF on myelin substrate, but not on laminin (Leibinger et al., 2012). The underlying cause for these disparate observations might be founded on the non-inhibitory nature of the fish optic nerve (Wanner et al., 1995). Similar to its effect in culture, rapamycin reduced, but not completely blocked axonal regrowth *in vivo* (Figure 6B). Remarkably, LIF induction after axotomy follows a similar time course as mTOR activation and functional recovery after optic nerve injury is similarly delayed upon LIF knockdown (Ogai et al., 2014). Therefore, it is quite conceivable that the partial inhibition of axonal regeneration upon rapamycin treatment reflects impaired PI3K/AKT/mTOR signaling, which, consistent with our *in vitro* results, would depend on LIF or other cytokine release shortly after axotomy (Figure 6B). Further experiments need to address this possibility in the future. As mTOR activity is quickly down-regulated after a short burst, it would be interesting to analyze whether prolonged activation of this pathway would lead to enhanced axon regeneration or whether the observed deactivation is essentially required for later regenerative processes. Due to the likely presence of intrinsic negative feedback loops within this pathway (Figure 6), these experiments might require inducible, RGC-specific expression of constitutively active mTOR mutants. Unfortunately, this approach is currently rather impractical in adult zebrafish.

Maintenance of pre-injury mTOR activity levels in injured mammalian RGCs, for instance by RGC-specific genetic deletion of PTEN, markedly promotes *in vivo* axon regeneration (Park et al., 2008; Smith et al., 2009). Likewise, constitutive activation of mTOR upon genetic deletion of TSC1 potently enhances axon regeneration of RGCs and DRG neurons (Park et al., 2008; Abe et al., 2010). On the other hand, the number of regenerating mouse RGC axons upon inflammatory stimulation was reduced by rapamycin treatment only at long distances, whereas the extent of short axons remained unaffected (Leibinger et al., 2012). These findings were interpreted in a way that mTOR is not essentially required for the initial switch of neurons into an active regenerative state. However, increased mTOR activity is seemingly important to maintain the regenerative state, potentially due to enhanced protein and lipid biosynthesis. In zebrafish, axon regeneration after optic nerve crush was significantly compromised, but not completely inhibited by rapamycin, suggesting a comparable ancillary role of mTOR in this regeneration-competent species.

Obviously, systemic rapamycin treatment could inhibit mTOR activity in cell types other than RGCs. For example, mTOR signaling has been shown to regulate oligodendrocyte differentiation and CNS myelination (Narayanan et al., 2009; Tyler et al., 2009) and the positive effects of rapamycin treatment after contusive spinal cord injury have been partly attributed to reduced secondary neural tissue damage due to suppression of macrophage/microglia activation and abrogation of reactive astrogliosis (Kanno et al., 2012). In addition, it is now known that chronic rapamycin treatment could inhibit mTORC2 just as the classic target mTORC1, thereby potentially affecting cytoskeleton dynamics (Sarbassov et al., 2006). However, it is currently unknown whether these processes/cell types might be involved in optic nerve regeneration in zebrafish. Nevertheless, the conclusion of a rather RGC-specific effect of our results is supported by the time course of mTOR activation and the differential functional recovery upon timed rapamycin treatment. Axon regeneration was only compromised in zebrafish receiving rapamycin at early time points after optic nerve crush (0–3 dpi), when mTOR was markedly induced in RGCs, whereas delayed rapamycin treatment (4–18 dpi) had no effect. Therefore, the induction of mTOR in cholinergic amacrines does not seem to contribute to optic nerve regeneration. Similarly, the observed reduction of regenerating axons upon rapamycin treatment could not be ascribed to impaired GFP marker expression. In contrast to mTOR-dependent induction of GAP43 in injured rodent sciatic nerves (Abe et al., 2010), no reduction of GAP43 promoter-driven gfp transcription was detected in zebrafish retinae upon rapamycin treatment. This result is in accordance with data showing no influence of rapamycin on the expression of GAP43 and other regeneration-associated genes in rodent retinae after inflammatory stimulation (Leibinger et al., 2012) and again indicates that inhibition of mTOR activity does not generally impair the transformation of RGCs into a regenerative state.

In conclusion, the current study demonstrates the differential regulation of mTOR activity in CNS regeneration-competent vs. -incompetent models. Although not required for basal axon growth, our data suggest that the early burst of mTOR activity still contributes to the positive regenerative outcome in zebrafish. Therefore, further mechanistic insights into functional axon regeneration are provided, highlighting that, at least in fish, other signaling pathways than mTOR are more crucially involved in CNS axon regeneration.

## Conflict of Interest Statement

The authors declare that the research was conducted in the absence of any commercial or financial relationships that could be construed as a potential conflict of interest.

## Abbreviations

4E-BP1: 4E-binding protein 1
AKT: protein kinase B
bpV: bisperoxovanadium
CHAT: cholinergic acetyl transferase
CNTF: ciliary neurotrophic factor
dpi: day(s) post injury
DRG: dorsal root ganglion
GAP43: growth associated protein 43
GFP: green fluorescent protein
JAK: Janus kinase
LIF: leukemia inhibitory factor
mTor: mechanistic target of rapamycin
PI3K: phosphatidylinositol 3-kinase
PTEN: phosphatase and tensin homolog
RGC: retinal ganglion cell
S6: ribosomal protein S6
S6K1: ribosomal protein S6 kinase 1
STAT: signal transducer and activator of transcription
TSC1: tuberous sclerosis complex 1.

## References

[B1] Abe N. Borson S. H. Gambello M. J. Wang F. Cavalli V. (2010). Mammalian target of rapamycin (mTOR) activation increases axonal growth capacity of injured peripheral nerves. J. Biol. Chem. 285, 28034–28043. 10.1074/jbc.M110.125336 20615870 PMC2934668

[B2] Becker C. G. Becker T. (2007). Growth and pathfinding of regenerating axons in the optic projection of adult fish. J. Neurosci. Res. 85, 2793–2799. 10.1002/jnr.21121 17131420

[B3] Bernhardt R. R. Tongiorgi E. Anzini P. Schachner M. (1996). Increased expression of specific recognition molecules by retinal ganglion cells and by optic pathway glia accompanies the successful regeneration of retinal axons in adult zebrafish. J. Comp. Neurol. 376, 253–264. 10.1002/(sici)1096-9861(19961209)376:2<253::aid-cne7>3.0.co;2-2 8951641

[B4] Bernhardt R. R. (1999). Cellular and molecular bases of axonal regeneration in the fish central nervous system. Exp. Neurol. 157, 223–240. 10.1006/exnr.1999.7059 10364435

[B5] Bormann P. Roth L. W. Andel D. Ackermann M. Reinhard E. (1999). zfNLRR, a novel leucine-rich repeat protein is preferentially expressed during regeneration in zebrafish. Mol. Cell. Neurosci. 13, 167–179. 10.1006/mcne.1999.0742 10328879

[B6] Cai D. Qiu J. Cao Z. McAtee M. Bregman B. S. Filbin M. T. (2001). Neuronal cyclic AMP controls the developmental loss in ability of axons to regenerate. J. Neurosci. 21, 4731–4739. 11425900 10.1523/JNEUROSCI.21-13-04731.2001 PMC6762375

[B7] Christie K. J. Webber C. A. Martinez J. A. Singh B. Zochodne D. W. (2010). PTEN inhibition to facilitate intrinsic regenerative outgrowth of adult peripheral axons. J. Neurosci. 30, 9306–9315. 10.1523/JNEUROSCI.6271-09.2010 20610765 PMC6632469

[B8] Diekmann H. Fischer D. (2013). Glaucoma and optic nerve repair. Cell Tissue Res. 353, 327–337. 10.1007/s00441-013-1596-8 23512141

[B9] Diekmann H. Kalbhen P. Fischer D. (2015). Characterization of optic nerve regeneration using transgenic zebrafish. Front. Cell. Neurosci. 9:118. 10.3389/fncel.2015.00118 25914619 PMC4391235

[B10] Duan X. Qiao M. Bei F. Kim I. J. He Z. Sanes J. R. (2015). Subtype-specific regeneration of retinal ganglion cells following axotomy: effects of osteopontin and mTOR signaling. Neuron 85, 1244–1256. 10.1016/j.neuron.2015.02.017 25754821 PMC4391013

[B11] Elsaeidi F. Bemben M. A. Zhao X. F. Goldman D. (2014). Jak/Stat signaling stimulates zebrafish optic nerve regeneration and overcomes the inhibitory actions of Socs3 and Sfpq. J. Neurosci. 34, 2632–2644. 10.1523/JNEUROSCI.3898-13.2014 24523552 PMC3921430

[B12] Fang Y. Park I. H. Wu A. L. Du G. Huang P. Frohman M. A.. (2003). PLD1 regulates mTOR signaling and mediates Cdc42 activation of S6K1. Curr. Biol. 13, 2037–2044. 10.1016/j.cub.2003.11.021 14653992

[B13] Fang Y. Vilella-Bach M. Bachmann R. Flanigan A. Chen J. (2001). Phosphatidic acid-mediated mitogenic activation of mTOR signaling. Science 294, 1942–1945. 10.1126/science.1066015 11729323

[B14] Fischer D. Leibinger M. (2012). Promoting optic nerve regeneration. Prog. Retin. Eye Res. 31, 688–701. 10.1016/j.preteyeres.2012.06.005 22781340

[B15] Fischer D. Pavlidis M. Thanos S. (2000). Cataractogenic lens injury prevents traumatic ganglion cell death and promotes axonal regeneration both *in vivo* and in culture. Invest. Ophthalmol. Vis. Sci. 41, 3943–3954. 11053298

[B16] Gao Y. Deng K. Hou J. Bryson J. B. Barco A. Nikulina E.. (2004). Activated CREB is sufficient to overcome inhibitors in myelin and promote spinal axon regeneration *in vivo*. Neuron 44, 609–621. 10.1016/j.neuron.2004.10.030 15541310

[B17] Goldberg J. L. Klassen M. P. Hua Y. Barres B. A. (2002). Amacrine-signaled loss of intrinsic axon growth ability by retinal ganglion cells. Science 296, 1860–1864. 10.1126/science.1068428 12052959

[B18] Harrington L. S. Findlay G. M. Lamb R. F. (2005). Restraining PI3K: mTOR signalling goes back to the membrane. Trends Biochem. Sci. 30, 35–42. 10.1016/j.tibs.2004.11.003 15653324

[B19] Hauk T. G. Leibinger M. Müller A. Andreadaki A. Knippschild U. Fischer D. (2010). Stimulation of axon regeneration in the mature optic nerve by intravitreal application of the toll-like receptor 2 agonist Pam3Cys. Invest. Ophthalmol. Vis. Sci. 51, 459–464. 10.1167/iovs.09-4203 19661221

[B20] Hirose K. Shiomi T. Hozumi S. Kikuchi Y. (2014). Mechanistic target of rapamycin complex 1 signaling regulates cell proliferation, cell survival and differentiation in regenerating zebrafish fins. BMC Dev. Biol. 14:42. 10.1186/s12861-014-0042-9 25480380 PMC4264545

[B21] Huang J. Manning B. D. (2009). A complex interplay between Akt, TSC2 and the two mTOR complexes. Biochem. Soc. Trans 37, 217–222. 10.1042/bst0370217 19143635 PMC2778026

[B22] Julien L. A. Roux P. P. (2010). mTOR, the mammalian target of rapamycin. Med. Sci. (Paris) 26, 1056–1060. 10.1051/medsci/201026121056 21187044

[B23] Kanno H. Ozawa H. Sekiguchi A. Yamaya S. Tateda S. Yahata K.. (2012). The role of mTOR signaling pathway in spinal cord injury. Cell Cycle 11, 3175–3179. 10.4161/cc.21262 22895182 PMC3466516

[B24] Laplante M. Sabatini D. M. (2009). mTOR signaling at a glance. J. Cell Sci. 122, 3589–3594. 10.1242/jcs.051011 19812304 PMC2758797

[B25] Leaver S. G. Cui Q. Bernard O. Harvey A. R. (2006). Cooperative effects of bcl-2 and AAV-mediated expression of CNTF on retinal ganglion cell survival and axonal regeneration in adult transgenic mice. Eur. J. Neurosci. 24, 3323–3332. 10.1111/j.1460-9568.2006.05230.x 17229081

[B26] Leibinger M. Andreadaki A. Fischer D. (2012). Role of mTOR in neuroprotection and axon regeneration after inflammatory stimulation. Neurobiol. Dis. 46, 314–324. 10.1016/j.nbd.2012.01.004 22273489

[B27] Leibinger M. Müller A. Andreadaki A. Hauk T. G. Kirsch M. Fischer D. (2009). Neuroprotective and axon growth-promoting effects following inflammatory stimulation on mature retinal ganglion cells in mice depend on ciliary neurotrophic factor and leukemia inhibitory factor. J. Neurosci. 29, 14334–14341. 10.1523/JNEUROSCI.2770-09.2009 19906980 PMC6665071

[B28] Leibinger M. Müller A. Gobrecht P. Diekmann H. Andreadaki A. Fischer D. (2013). Interleukin-6 contributes to CNS axon regeneration upon inflammatory stimulation. Cell Death Dis. 4:e609. 10.1038/cddis.2013.126 23618907 PMC3641349

[B29] Li X. Yang Q. Yu H. Wu L. Zhao Y. Zhang C.. (2014). LIF promotes tumorigenesis and metastasis of breast cancer through the AKT-mTOR pathway. Oncotarget 5, 788–801. 24553191 10.18632/oncotarget.1772 PMC3996668

[B30] Lindsey A. E. Powers M. K. (2007). Visual behavior of adult goldfish with regenerating retina. Vis. Neurosci. 24, 247–255. 10.1017/s0952523806230207 17592671

[B31] Lingor P. Tönges L. Pieper N. Bermel C. Barski E. Planchamp V.. (2008). ROCK inhibition and CNTF interact on intrinsic signalling pathways and differentially regulate survival and regeneration in retinal ganglion cells. Brain 131, 250–263. 10.1093/brain/awm284 18063589

[B32] Liu Q. Londraville R. L. (2003). Using the adult zebrafish visual system to study cadherin-2 expression during central nervous system regeneration. Methods Cell Sci. 25, 71–78. 10.1023/b:mics.0000006854.18378.fc 14739590

[B33] Liu K. Lu Y. Lee J. K. Samara R. Willenberg R. Sears-Kraxberger I.. (2010). PTEN deletion enhances the regenerative ability of adult corticospinal neurons. Nat. Neurosci. 13, 1075–1081. 10.1038/nn.2603 20694004 PMC2928871

[B34] Livak K. J. Schmittgen T. D. (2001). Analysis of relative gene expression data using real-time quantitative PCR and the 2(-Delta Delta C(T)) Method. Methods 25, 402–408. 10.1006/meth.2001.1262 11846609

[B35] Ma L. Chen Z. Erdjument-Bromage H. Tempst P. Pandolfi P. P. (2005). Phosphorylation and functional inactivation of TSC2 by Erk implications for tuberous sclerosis and cancer pathogenesis. Cell 121, 179–193. 10.1016/j.cell.2005.02.031 15851026

[B36] Maiese K. Chong Z. Z. Shang Y. C. Wang S. (2013). mTOR: on target for novel therapeutic strategies in the nervous system. Trends Mol. Med. 19, 51–60. 10.1016/j.molmed.2012.11.001 23265840 PMC3534789

[B37] Manning B. D. Cantley L. C. (2007). AKT/PKB signaling: navigating downstream. Cell 129, 1261–1274. 10.1016/j.cell.2007.06.009 17604717 PMC2756685

[B38] McDowell A. L. Dixon L. J. Houchins J. D. Bilotta J. (2004). Visual processing of the zebrafish optic tectum before and after optic nerve damage. Vis. Neurosci. 21, 97–106. 10.1017/s0952523804043019 15259561

[B39] Mensinger A. F. Powers M. K. (2007). Visual function in regenerating teleost retina following surgical lesioning. Vis. Neurosci. 24, 299–307. 10.1017/s0952523807070265 17550640

[B40] Müller A. Hauk T. G. Leibinger M. Marienfeld R. Fischer D. (2009). Exogenous CNTF stimulates axon regeneration of retinal ganglion cells partially via endogenous CNTF. Mol. Cell. Neurosci. 41, 233–246. 10.1016/j.mcn.2009.03.002 19332123

[B41] Narayanan S. P. Flores A. I. Wang F. Macklin W. B. (2009). Akt signals through the mammalian target of rapamycin pathway to regulate CNS myelination. J. Neurosci. 29, 6860–6870. 10.1523/JNEUROSCI.0232-09.2009 19474313 PMC2757755

[B42] Ning K. Drepper C. Valori C. F. Ahsan M. Wyles M. Higginbottom A.. (2010). PTEN depletion rescues axonal growth defect and improves survival in SMN-deficient motor neurons. Hum. Mol. Genet. 19, 3159–3168. 10.1093/hmg/ddq226 20525971

[B43] Ogai K. Kuwana A. Hisano S. Nagashima M. Koriyama Y. Sugitani K.. (2014). Upregulation of leukemia inhibitory factor (LIF) during the early stage of optic nerve regeneration in zebrafish. PLoS One 9:e106010. 10.1371/journal.pone.0106010 25162623 PMC4146584

[B44] Park K. K. Liu K. Hu Y. Smith P. D. Wang C. Cai B.. (2008). Promoting axon regeneration in the adult CNS by modulation of the PTEN/mTOR pathway. Science 322, 963–966. 10.1126/science.1161566 18988856 PMC2652400

[B45] Pernet V. Joly S. Dalkara D. Jordi N. Schwarz O. Christ F.. (2013). Long-distance axonal regeneration induced by CNTF gene transfer is impaired by axonal misguidance in the injured adult optic nerve. Neurobiol. Dis. 51, 202–213. 10.1016/j.nbd.2012.11.011 23194670

[B46] Posner B. I. Faure R. Burgess J. W. Bevan A. P. Lachance D. Zhang-Sun G.. (1994). Peroxovanadium compounds. A new class of potent phosphotyrosine phosphatase inhibitors which are insulin mimetics. J. Biol. Chem. 269, 4596–4604. 8308031

[B47] Sarbassov D. D. Ali S. M. Sengupta S. Sheen J. H. Hsu P. P. Bagley A. F.. (2006). Prolonged rapamycin treatment inhibits mTORC2 assembly and Akt/PKB. Mol. Cell 22, 159–168. 10.1016/j.molcel.2006.03.029 16603397

[B48] Schmid A. C. Byrne R. D. Vilar R. Woscholski R. (2004). Bisperoxovanadium compounds are potent PTEN inhibitors. FEBS Lett. 566, 35–38. 10.1016/s0014-5793(04)00435-1 15147864

[B49] Schwab M. E. Bartholdi D. (1996). Degeneration and regeneration of axons in the lesioned spinal cord. Physiol. Rev. 76, 319–370. 8618960 10.1152/physrev.1996.76.2.319

[B50] Smith P. D. Sun F. Park K. K. Cai B. Wang C. Kuwako K.. (2009). SOCS3 deletion promotes optic nerve regeneration *in vivo*. Neuron 64, 617–623. 10.1016/j.neuron.2009.11.021 20005819 PMC2796263

[B51] Stuermer C. A. Bastmeyer M. Bähr M. Strobel G. Paschke K. (1992). Trying to understand axonal regeneration in the CNS of fish. J. Neurobiol. 23, 537–550. 10.1002/neu.480230508 1431836

[B52] Tyler W. A. Gangoli N. Gokina P. Kim H. A. Covey M. Levison S. W.. (2009). Activation of the mammalian target of rapamycin (mTOR) is essential for oligodendrocyte differentiation. J. Neurosci. 29, 6367–6378. 10.1523/JNEUROSCI.0234-09.2009 19439614 PMC2827328

[B53] Udvadia A. J. (2008). 3.6 kb genomic sequence from Takifugu capable of promoting axon growth-associated gene expression in developing and regenerating zebrafish neurons. Gene Expr. Patterns 8, 382–388. 10.1016/j.gep.2008.05.002 18599366 PMC2603057

[B54] Wanner M. Lang D. M. Bandtlow C. E. Schwab M. E. Bastmeyer M. Stuermer C. A. (1995). Reevaluation of the growth-permissive substrate properties of goldfish optic nerve myelin and myelin proteins. J. Neurosci. 15, 7500–7508. 7472501 10.1523/JNEUROSCI.15-11-07500.1995 PMC6578052

[B55] Westerfield M. (1989). The zebrafish book: a guide for the laboratory use of zebrafish (Brachydanio rerio). Eugene: University of Oregon.

